# Comparison of 8 weeks standard treatment (rifampicin plus clarithromycin) *vs.* 4 weeks standard plus amoxicillin/clavulanate treatment [RC8 *vs.* RCA4] to shorten Buruli ulcer disease therapy (the BLMs4BU trial): study protocol for a randomized controlled multi-centre trial in Benin

**DOI:** 10.1186/s13063-022-06473-9

**Published:** 2022-07-08

**Authors:** Roch Christian Johnson, Emma Sáez-López, Esaï Sèdjro Anagonou, Godwin Gérard Kpoton, Adjimon Gilbert Ayelo, Ronald Sètondji Gnimavo, Franck Zinsou Mignanwande, Jean-Gabin Houezo, Ghislain Emmanuel Sopoh, Juliet Addo, Lindsay Orford, Georgios Vlasakakis, Nandita Biswas, Felix Calderon, Oscar Della Pasqua, Anna Gine-March, Zaida Herrador, Alfonso Mendoza-Losana, Gabriel Díez, Israel Cruz, Santiago Ramón-García

**Affiliations:** 1 Centre Interfacultaire de Formation et de Recherche en Environnement pour le Développement Durable (CIFRED), Université d’Abomey-Calavi, Abomey-Calavi, Benin; 2 Fondation Raoul Follereau, Paris, France; 3 Department of Microbiology, Faculty of Medicine, University of Zaragoza, Zaragoza, Spain; 4 CIBER Enfermedades Respiratorias (CIBERES), Instituto de Salud Carlos III, Madrid, Spain; 5 Programme National de Lutte Contre l’Ulcère de Buruli, Cotonou, Bénin; 6 Institut Régional de Santé Publique, Université d’Abomey-Calavi, Ouidah, Bénin; 7 Global Health Catalyst, GlaxoSmithKline, Brentford, London, UK; 8 Research, CPMS, GlaxoSmithKline, Brentford, London, UK; 9 Clinical Pharmacology & Therapeutics Group, University College London, London, UK; 10 Consiglio Nazionale Delle Ricerche, Istituto Per Le Applicazioni del Calcolo, Rome, Italy; 11 Anesvad Foundation, Bilbao, Spain; 12 National Centre for Epidemiology, Instituto de Salud Carlos III, Madrid, Spain; 13 Present Address: Department of Bioengineering and Aeroespace Engineering, Carlos III University of Madrid, Madrid, Spain; 14 National School of Public Health, Instituto de Salud Carlos III, Madrid, Spain; 15 CIBER Enfermedades Infecciosas (CIBERINFEC), Instituto de Salud Carlos III, Madrid, Spain; 16 Research & Development Agency of Aragon (ARAID) Foundation, Zaragoza, Spain

**Keywords:** Buruli ulcer, Skin neglected tropical disease, Treatment shortening, Non-inferiority, Drug combination, Amoxicillin, Clavulanate, Pharmacokinetics, Bacterial clearance

## Abstract

**Background:**

Buruli ulcer (BU) is a neglected tropical disease caused by *Mycobacterium ulcerans* that affects skin, soft tissues, and bones, causing long-term morbidity, stigma, and disability. The recommended treatment for BU requires 8 weeks of daily rifampicin and clarithromycin together with wound care, physiotherapy, and sometimes tissue grafting and surgery. Recovery can take up to 1 year, and it may pose an unbearable financial burden to the household.

Recent in vitro studies demonstrated that beta-lactams combined with rifampicin and clarithromycin are synergistic against *M. ulcerans*. Consequently, inclusion of amoxicillin/clavulanate in a triple oral therapy may potentially improve and shorten the healing process.

The BLMs4BU trial aims to assess whether co-administration of amoxicillin/clavulanate with rifampicin and clarithromycin could reduce BU treatment from 8 to 4 weeks.

**Methods:**

We propose a randomized, controlled, open-label, parallel-group, non-inferiority phase II, multi-centre trial in Benin with participants stratified according to BU category lesions and randomized to two oral regimens: (i) Standard: rifampicin plus clarithromycin therapy for 8 weeks; and (ii) Investigational: standard plus amoxicillin/clavulanate for 4 weeks. The primary efficacy outcome will be lesion healing without recurrence and without excision surgery 12 months after start of treatment (i.e. cure rate). Seventy clinically diagnosed BU patients will be recruited per arm. Patients will be followed up over 12 months and managed according to standard clinical care procedures. Decision for excision surgery will be delayed to 14 weeks after start of treatment. Two sub-studies will also be performed: a pharmacokinetic and a microbiology study.

**Discussion:**

If successful, this study will create a new paradigm for BU treatment, which could inform World Health Organization policy and practice. A shortened, highly effective, all-oral regimen will improve care of BU patients and will lead to a decrease in hospitalization-related expenses and indirect and social costs and improve treatment adherence. This trial may also provide information on treatment shortening strategies for other mycobacterial infections (tuberculosis, leprosy, or non-tuberculous mycobacteria infections).

**Trial registration:**

ClinicalTrials.gov NCT05169554. Registered on 27 December 2021.

**Supplementary Information:**

The online version contains supplementary material available at 10.1186/s13063-022-06473-9.

## Background

Buruli ulcer (BU) is a chronic debilitating skin and soft tissue mycobacterial infection that without treatment frequently progresses to massive ulceration. The greatest burden falls on children below 15 years in sub-Saharan Africa, accounting for at least 50% of those affected by BU [1]. In 2019, Africa presented the highest number of cases (86.2%) among the new reported cases (2260) [2]. This skin neglected tropical disease (NTD) generally affects poor communities in remote rural areas with limited access to health services. Although mortality is low, permanent disfigurement and disability are high, occurring in up to 25% of the cases [3].

BU is caused by *Mycobacterium ulcerans* but transmission patterns remain unknown, and thereby, early diagnosis and treatment are crucial to minimize morbidity costs and prevent long-term disability. Before 2004, surgical excision and skin grafting remained the mainstay of BU treatment [4–6] until clinical evidence showed the effectiveness of rifampicin-streptomycin [7]. Serious adverse events (SAEs) associated with the injectable streptomycin and the lack of an efficacious oral treatment, however, remained one of the main obstacles to decentralizing care in rural areas. Since 2017, the World Health Organization (WHO) recommends an 8-week completely oral daily combination therapy of rifampicin-clarithromycin (RIF/CLA) [8]. However, access to medicines is difficult, and the need of hospitalization for treatment impacts a patient’s household income and compromises adherence to the 8-week antibiotic course [9–11]. Similar to tuberculosis (TB), RIF is the cornerstone drug for BU therapy, showing a direct relation between dose increase and therapy efficacy [12] due to its bactericidal and sterilizing activity [13]. High-dose RIF studies suggest that BU treatment could be shortened if RIF dose was increased [13, 14]. Nevertheless, this approach raises concerns due to high-dose RIF-related hepatotoxicity and feasibility of its implementation in the clinical practice. It would be thus desirable to maintain the current dose and improve RIF efficacy without compromising tolerability and toxicity; this could be achieved using RIF in combination with synergistic partners [15–17]. Recent studies from partners of this Consortium showed in in vitro studies that beta-lactams strongly increased the activity of RIF and CLA against *M. ulcerans* [16]. These observations were further confirmed by time-­kill kinetics (*unpublished data, manuscript under preparation, BLMs4BU Consortium*).

Among all beta-lactams, we focused on amoxicillin/clavulanate (AMX/CLV), which is oral, suitable for the treatment of children, pregnant women, and adults and with a long track record of clinical efficacy and safety [18]. Due to its rapid bactericidal activity, AMX/CLV would be effective at reducing initial bacterial burden, paradoxical responses [19], local levels of the immune-suppressive mycolactone toxin, and allowing recovery of the host immune response to clear remaining bacteria [14]. Furthermore, the sterilizing activity of synergistic combinations of beta-lactams and RIF could target the remaining persistent populations ultimately allowing for a potentially shorter duration of treatment and healing periods [15, 20].

A shortened, highly effective, all-oral regimen based on already approved drugs is thus urgently needed to improve care for this NTD by reducing both duration of treatment and time to healing for all type of lesions after therapy completion. This will ultimately reduce the associated indirect and social costs, and barriers to access therapy. Moreover, developing more effective and accessible NTD therapeutic strategic will up thrust the lifespan and lifestyle of the socioeconomically deprived areas affected by this disease. Therefore, in this study, we propose the combination of AMX/CLV with current oral BU therapy, RIF/CLA, as a new BU therapy with the potential of treatment shortening and ready implementation in the field. For this purpose, a Phase II clinical trial will be conducted in Benin.

### Aims of the study

#### Primary objective

The primary objective is to demonstrate the non-inferiority of 4-week co-administration of AMX/CLV with RIF/CLA compared to the standard 8-week RIF/CLA in cure rates at 12 months post initiation of treatment, thus supporting reduction of BU treatment from 8 to 4 weeks.

#### Secondary objectives

1. To test the hypothesis that 4-week combination therapy of RIF plus CLA plus AMX/CLV (RCA4) could improve the median bacterial clearance rate over the currently WHO-recommended 8-week RIF plus CLA (RC8) therapy,
2. To investigate whether a hypothetical increase in the median bacterial clearance rate due to the addition of AMX/CLV correlates with a reduction in the median time to healing of the BU lesions,
3. To explore the effect of AMX/CLV on RIF/CLA pharmacokinetics (PK), and
4. To characterize the population PK (POPPK) of RIF and AMX in BU patients randomized in the investigational regimen (RCA4).

### Methods/design

#### Study design

BLMs4BU study is a Phase II, randomized, controlled, open-label, parallel-group, non-inferiority, multi­centre trial, with two treatment arms:

- Standard [RC8]: RIF plus CLA (RC) therapy for 8 weeks; and
- Investigational [RCA4]: standard (RC) plus AMX/CLV (A) for 4 weeks.

**Fig. 1:**
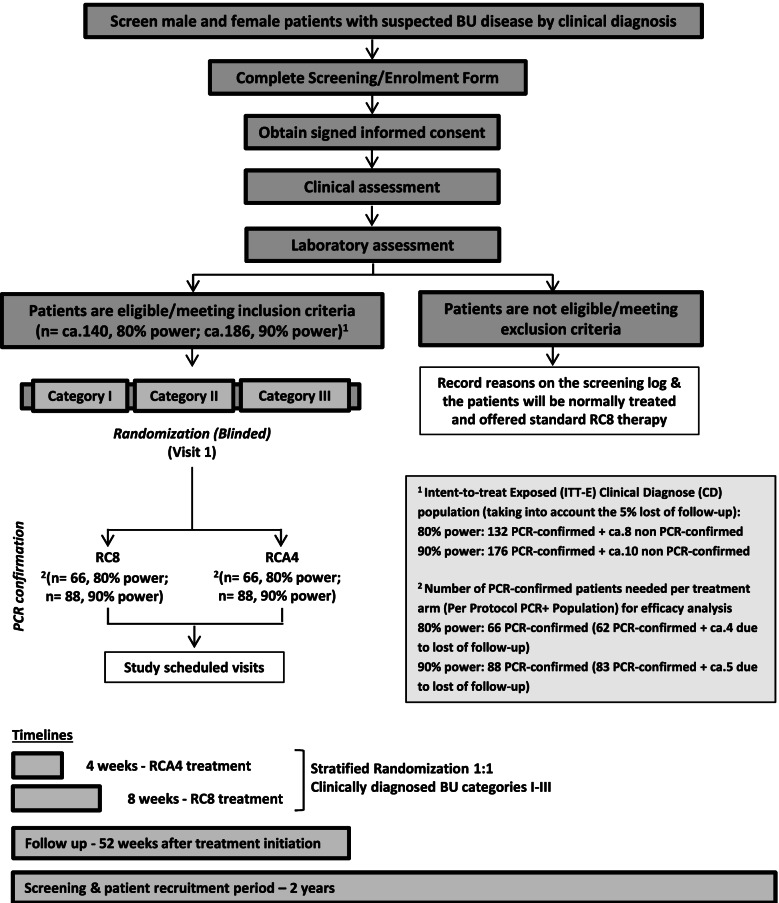
Flow chart of sampling, recruitment and timelines of the clinical trial

**Fig. 2:**
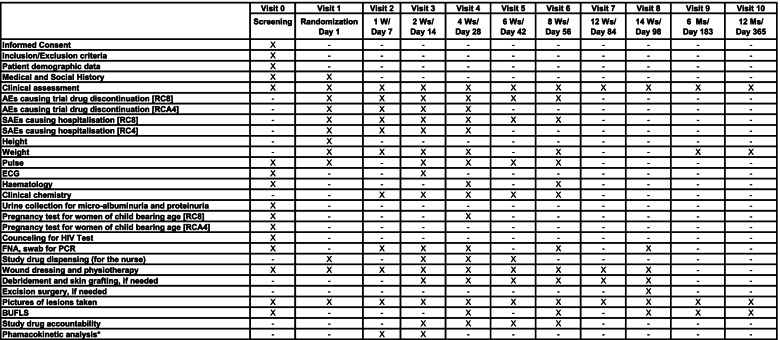
Schedule of screening, randomization, interventions and assessment of the patients in the BLMs4BU study. AEs, adverse events; BUFLS, Buruli Ulcer Functional Limitation Score; FNA, fine-needle aspiration; M, month; HIV, human immunodeficiency virus; PCR, polymerase chain reaction; RC8, 8 weeks of rifampicin-clarithromycin; RCA4, 4 weeks of rifampicin-clarithromycin plus amoxicillin/clavulanate; SAEs, serious adverse events; W, week. *PK analysis (blood samples). It will be performed between days 7 and 14 after starting the treatment, prior to the second daily dose of CLA and AMX/CLV. Sampling times will include a pre-dose at time 0 (within 10 min pre-dose) and at times 0.5, 1, 1.5, 2, 2.5, 3, 5, 7.5, and 10 h post-dose

The flow chart of the clinical trial study design and timelines are depicted in Figs. 1 and 2. In addition, a PK and microbiology study will be also performed in a complementary manner. Details of ethics approval are provided at the end of the manuscript.

#### Study setting

**Fig. 3:**
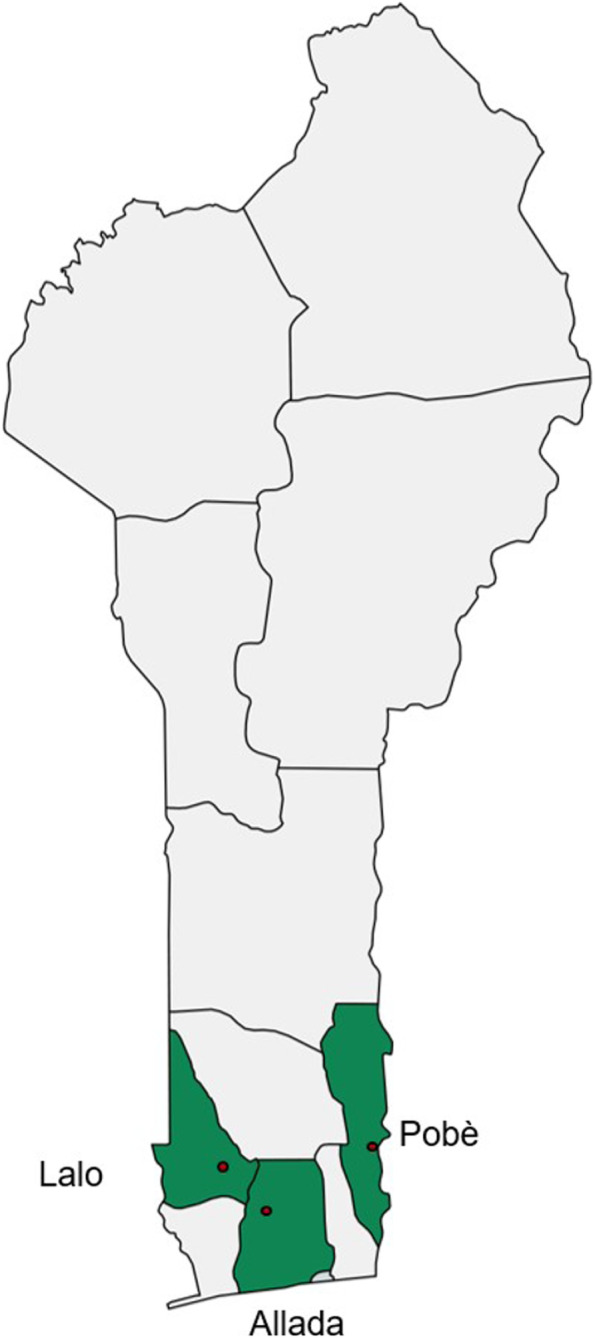
Location of the Lalo, Allada, and Pobè clinical sites in Benin, where the study will be conducted. Figure made with mapchart.net [21]

The main study will be conducted in three clinical sites that are Centers of Detection and Treatment of Buruli Ulcer (Centre de Dépistage et de Traitement de l’Ulcère de Buruli, CDTUB) in Benin, located in the towns of Pobè, Allada and Lalo, in the south of the country, where BU is endemic (Fig. 3). PK and bacterial clearance sub-studies will be performed only at the Pobè treatment centre.

#### Eligibility criteria

**Fig. 4:**
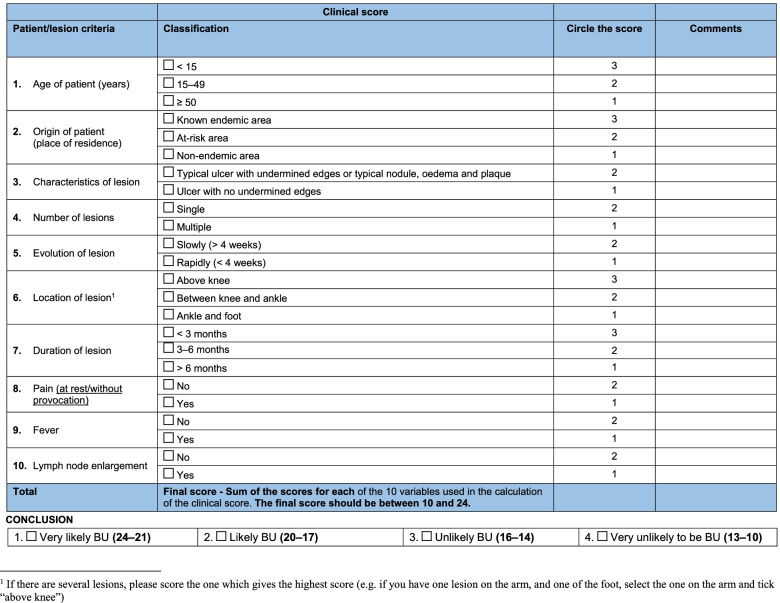
WHO clinical diagnosis scoring criteria

All patients (both genders) aged ≥ 5 years and ≤ 70 years with a very likely or likely new clinical diagnosis of BU according to WHO scoring criteria (Fig. 4) [22–24] (all categories: I, II, III) and normal electrocardiogram (ECG) at baseline. Patients meeting inclusion criteria will be invited to participate and enrolled in the study. Non-inclusion criteria are listed in Table 1.

**Table 1:** Overview of inclusion and exclusion criteria in the BLMs4BU study

Inclusion criteria
Patients must fulfil the following criteria:
•Age of ≥ 5 years and adults ≤ 70 years
•New clinical diagnosis of BU (all categories: I, II, and III)
•Normal ECG
**Exclusion criteria**
Patients with any of the following criteria are not eligible:
•Children < 5 years and adults > 70 years
•Children in foster care
•Patients weighing less than 11 kg
•Pregnancy positive (urine test: beta-HCG positive)
•Previous treatment of Buruli ulcer, tuberculosis, or leprosy with at least one of the study drugs
•Patients with diagnosed leprosy or tuberculosis disease
•Hypersensitivity to at least one of the study drugs or to any of the excipients
•History of a severe immediate hypersensitivity reaction (e.g. anaphylaxis) to another beta-lactam agent (e.g. a cephalosporin, carbapenem, or monobactam)
•History of jaundice/hepatic impairment due to amoxicillin/clavulanic acid or rifampicin
•Patients with history of treatment with macrolide or quinolone antibiotics, anti-tuberculosis medication, or immuno-modulatory drugs including corticosteroids within 1 month
•Patients currently receiving treatment with any drugs likely to interact with the study medications, i.e. anticoagulants, cyclosporine, phenytoin, or phenobarbitone. Users of oral contraceptives should be notified that such contraceptive is less reliable if taken with rifampicin; additional (mechanical) contraceptive methods will be discussed with the study participant
•Patients with HIV co-infection
•Patients with QTc prolongation > 450 ms on ECG or on other medication known to prolong the QTc interval. In this case, if suspected of BU disease, patients will be offered 8-week rifampicin plus streptomycin therapy
•Patients unable to take oral medication or having gastrointestinal disease likely to interfere with drug absorption
•Patients with history or having current clinical signs of ascites, jaundice, myasthenia gravis, renal dysfunction (known or suspected), diabetes mellitus, and severe immune compromise, or evidence of tuberculosis, or leprosy; terminal illness (e.g. metastasized cancer), haematological malignancy, chronic liver disease, abnormal liver function test, and coronary artery disease, or any other condition that would preclude enrolment into the study in the study physician’s opinion
•Evidence of a clinically significant (as judged by the Investigator) condition or abnormality (other than the indication being studied) that might compromise safety or the interpretation of trial efficacy or safety endpoints
•Patients with known or suspected bowel strictures who cannot tolerate clarithromycin
•Patients with a mental health condition that is likely to interfere with compliance with the study protocol in the opinion of the study physician
•Patients (or parent/legal representative) who are not willing to give informed consent or withdrawal of consent
•Specific exclusion criteria for the PK sub-study are patients less than 15 years old or less than 40 kg or with renal impairment with a creatinine level higher than the normal one in Benin (7–14 mg/L)
•Patients who cannot confirm the absence of previous BU treatment or with potential difficulties to be followed up

### Procedures and measurements

#### Screening and informed consent

Information, education, and communication campaigns and active case finding will be performed weekly in the communities during the study period together with mobile medical consultations of skin diseases which will be implemented too. These activities which will involve community volunteers, teachers, nurses from decentralized centres, and traditional healers, and they will help for detection of BU cases and referral to the BU health centres. Patients suspected to have BU will be recruited from villages covered by the different study sites participating in this trial: Pobè, Lalo, and Allada. During health promotion activities, people living in endemic villages will be encouraged to participate at workshops where information will be provided beforehand of the details and intentions of the study, and the benefits it might bring to them and the community at large. Culturally sensitive aspects of the protocol, especially testing for pregnancy and co-infection with human immunodeficiency virus (HIV) or TB will be discussed with community leaders in the villages.

Potential study participants will receive a unique study code number, kept with the site clinical investigator and the clinical study coordinators. They will also be informed about the importance of early reporting as well as their continuing participation to the end of the study.

At the study site, lesions will be clinically examined for compatibility with BU and scored according to WHO recommendations (Fig. 4) [22, 23] through consensus by the clinical investigators in each study site. Patients will also be assessed for eligibility by the site clinician, or health care worker delegate. If the lesion is clinically diagnosed as a very likely or likely BU lesion by the clinicians at the trial sites, after verbal and written explanation appropriate consent/assent by participants, and/or consent by their caregivers or legal representatives will be sought. The patient will be enrolled into the study only after informed consent has been obtained and forms are signed by both the study team member (Clinical Principal Investigator or deputy) and the study participant (sign or thumbprint, in case the participant is illiterate witnessed by an independent observer who is literate and is not part of the clinical trial team).

#### Assessment of the lesions

A full description of all lesions (including stage: ulcer, non-ulcer; lesion size and category; digital photography) will be obtained by the health care professional. The maximum diameter and the diameter at right angles to the maximum diameter line of the lesions will be measured using a transparent ruler, which will be disinfected between patients. The margins of the indurated area of the lesion determined by palpation and marked by a non-permanent marker will form the boundaries to be measured. In ulcerative lesions, these will be the margins of the skin defect and the indurated area. Digital photographs will be taken of all lesions as part of the documentation of clinical evolution. In addition, a questionnaire will be filled in to determine the Buruli Ulcer Functional Limitation Score (BUFLS) [24–28].

#### Laboratory evaluation

Blood and urine samples will be collected and laboratory analysis performed at the clinical sites. The specimens will be collected, transported, stored, and prepared according to local protocols. Blood will be stored at 2–8 °C before handling within the required time. Urine samples will be transported at room temperature and must be handled within 2 h after collection. If necessary, samples will be eventually destroyed by incineration.

The following tests will be performed: routine blood testing (clinical haematology including haemoglobin, white blood cells, and red blood cells; clinical chemistry in serum); urine test (routine urinalysis); blood HIV-testing (following counselling and once consent for HIV-testing is provided); assessment of the corrected QT (QTc) prolongation on ECG and; urine beta-human chorionic gonadotropin levels (hCG) for all females of child-bearing age (10–50 years) to rule out pregnancy.

Pregnancy test and HIV counselling and testing will be treated with strict confidentiality. Patients with a positive pregnancy test at screening will be not be enrolled in the study. The test result will firstly be discussed with the study participant (even if this person is below the age of 18 years) and, if agreed, with the parent or caretaker. The pregnancy test will be repeated at week 4 for all women of child-bearing age randomized in the RC8 group. If in this case the pregnancy test demonstrates pregnancy, the patient will be excluded from further participation in the trial but will continue on WHO-recommended antibiotic therapy and regular wound care follow-up procedures, according to the BU National Program (PNLLUB) guidelines for management of BU. Data collected prior to this will however be included in the analysis.

In addition, an aliquot of 2 mL of serum will be stored (− 20 °C) for future diagnostic test development and evaluation at treatment sites. Specific consent will be obtained for this. Samples will be stored anonymously at a central location for a minimum of 5 years and a maximum of 10 years after completion of the study, after which these specimens will be destroyed according to local guidelines and protocols. The potential use of the stored samples is included in the informed consent form. Nevertheless, further use of the samples will need to be approved by the Ethics Committee (EC) in Benin.

#### Randomization

Each patient will be assigned a unique study code number, kept by the site Clinical PI and the clinical site study coordinators. Data relevant for randomization will be sent by email to the randomization manager at the Data Management and Monitoring Centre (DMMC) at Instituto de Salud Carlos III (ISCIII). The DMMC team generates the random sequence upon receiving the information from the clinical sites, i.e. patient code and category of the lesion. Participants are randomly assigned to either control or experimental group as per a computer-generated randomization schedule stratified by site using permuted blocks of random sizes (*n* = 4). Blocked randomization assures generation of study groups with the same size, and a close balance on the numbers in each group by site at any time during the trial. The random sequence is generated in an Excel spreadsheet via Random.org.

Data for randomization will be sent by text (SMS) where it is not possible to send by email. After computer-generated random allocation to either RC8 or RCA4, the randomization group will be provided by email to the site Clinical PI and the clinical site study coordinators. Randomization will be stratified by lesion categories I, II, or III, using a fixed block size within each strata. The target sample size is based on confirmation of *M. ulcerans* infection by polymerase chain reaction (PCR), which will be assessed after the randomization process.

#### Type of data collected

The data to be collected from study participants will include stage and presentation of the disease, i.e. ulcer, non-ulcer; lesion size and category; digital photograph of the lesion, study site; and demographic data. The BU01.N form will be completed according to the WHO recommendations [24]. All data will be recorded in the Case Report Form (CRF) for every patient.

#### Data sharing and access

We will generate a metadata record describing all datasets arising from the project and make this available through an appropriate repository (e.g. DRYAD https://datadryad.org/). This will act as a site for long-term curation and preservation. Scientific reports will be written and submitted to peer-reviewed journals during and at the end of the project. Data underpinning these articles will be made available for access.

#### Microbiological analysis

Diagnostic procedures will depend on the presentation of the disease. In non-ulcerated lesions, fine-needle aspiration (FNA) will be the usual sampling procedure with two samples taken per lesion. In ulcerated lesions, two cotton wool swabs from undermined ulcer edges will be taken.

All sample collection procedures will follow WHO guidelines [29]. For the purpose of this clinical study, all specimens will be examined for *M. ulcerans* confirmation by PCR using insertion sequence IS*2404* and direct microscopy for acid-fast bacilli (AFB) [30]. In addition, microbiology cultures and drug susceptibility testing, including the assessment of synergistic drug interactions in clinical isolates, will also be performed as part of this trial at baseline and in the case of relapse of unhealed lesions to test for possible development of resistance or loss of synergy.

#### Treatment

Two treatment regimens will be used in this clinical study protocol (Table 2):

- WHO-recommended standard (control): 8 weeks of rifampicin plus clarithromycin [8].
- Investigational: 4 weeks of rifampicin plus clarithromycin plus amoxicillin/clavulanate.

**Table 2:** Clinical trial treatment cohort regimens

Treatment cohort	Dose	Posology	Treatment length
Standard arm: [RC8]	^**1**^Rifampicin 600 mg	Daily	8 weeks
	^**1**^Clarithromycin 500 mg	Twice daily	8 weeks
Investigational arm: [RCA4]	^**1**^Rifampicin 600 mg	Daily	4 weeks
	^**1**^Clarithromycin 500 mg	Twice daily	4 weeks
	^**2**^Amoxicillin/clavulanate 1000/125 mg	Twice daily	4 weeks

^1^Dose indicated are for a 60 kg adult and will be standardized according to patient body weight following the WHO guidelines [31]. Rifampicin (R), 10 mg/kg; Clarithromycin (C) 7.5 mg/kg

^2^Dose of amoxicillin/clavulanate (A) 1000/125 mg twice daily, which makes a total of 2000/250 mg/day, for patients over 40 kg, and 22.5/5.6 mg/kg twice daily, which makes a total of 45/11.25 mg/kg/day, for those equal and below 40 kg. For children, posology will be adapted to the age of the patient according to drug manufacturer indications. There are no current recommendations for the use of amoxicillin/clavulanate for BU infections. Those indicated above are recommended doses for complicated infections according to manufacturer indications

During the trial period of antibiotic treatment, patients will be either admitted to the hospital, or receive directly observed therapy at one of the participating satellite health centres. All RC8 or RCA4 treatment will be fully supervised by health care staff to avoid possible side effects (Table 3).

**Table 3:** List of possible effects due to rifampicin, clarithromycin, and amoxicillin/clavulanate

	**Rifampicin**	**Clarithromycin**	**Amoxicillin/clavulanate**
More common	None	None	Nausea, vomiting, headache, diarrhoea, gas, stomach pain, skin rash or itching, white patches in your mouth or throat
Less common/rare	Chills, difficult breathing, dizziness, fever, headache, itching, muscle and bone pain, shivering, skin rash and redness; bloody or cloudy urine, increased frequency of urination or amount of urine, loss of appetite, nausea or vomiting, sore throat, unusual bleeding or bruising, unusual tiredness or weakness, yellow eyes or skin	Loss of appetite, nausea, dyspepsia; changes in taste and smell sensation; headache; ringing or buzzing in the ears (tinnitus), dizziness; liver enzyme elevation; allergic skin reactions including Stevens-Johnson syndrome; blood dyscrasias; reversible loss of hearing	Watery or bloody diarrhoea; pale or yellowed skin, dark coloured urine, crystalluria, fever, confusion or weakness; easy bruising or bleeding; skin rash, bruising, severe tingling, numbness, pain, muscle weakness; agitation, confusion, unusual thoughts or behaviour, seizures (convulsions); nausea, upper stomach pain, itching, loss of appetite, clay-coloured stools, jaundice (yellowing of the skin or eyes), or severe skin reaction; sore throat, swelling in face or tongue, burning in eyes, skin pain, followed by a red or purple skin rash that spreads (especially in the face or upper body) and causes blistering and peeling

Adverse events will be registered and coded for further reporting following MedDRA guidelines.

#### Follow-up

Follow-up will be according to the visit schedule (Fig. 2) and will take place at the clinical sites, outpatients clinic, and/or in the communities. Patients missing clinic appointments will be followed up in the community by the study team. This information and the reasons for non-attendance will be noted in the patient’s file in order to implement strategies and measures to prevent future loss to follow-up.

For visits outside the scheduled ones, visits that are early in the follow-up line (visits scheduled up to week 14), a ± 7 days window can be accepted, and a ± 14 days window for follow-up visits which are late in the follow-up line (visits at 6 and 12 months), without a protocol deviation being reported. In both scenarios, the days are counted from the day (before or after, depending on the case) for which the visit was scheduled. If the time of the visit is outside this protocol window, data can still be collected; however, a protocol deviation should be reported.

Patients will always be followed up by local clinicians (in a non-blinded manner) as they usually do in normal BU care. Patients will have clinical assessment of the lesions and scars at every visit or any time the patient self-reports (any reason), or a paradoxical reaction is suspected/diagnosed. Local clinicians will take any decisions regarding wound debridement and skin grafting, according to standard care procedures. Any decision for excision surgery will be delayed to 14 weeks after initiation of antibiotic treatment according to recent findings showing that BU patients benefited from delaying the decision to operate from 8 to 14 weeks [32]. In addition, a clinical expert panel (Technical Expert Panel, TEP), blinded to treatment allocation in order to make objectives comparisons, will assess the need of excision surgery in both treatment arms. Their assessment will be conferred with that of the local clinicians.

#### Study schedule

Study set up will last 12 months, whereas recruitment is estimated to last 24 months. Hence, last patient, last visit (LPLV) will occur 36 months after the first patient is randomized. Study close out will be 6 months after LPLV.

#### Discontinuation from the study medication

Patients who meet study eligibility criteria, give informed consent (or assent plus consent by caretakers) but who may not have completed the full treatment course of antibiotic therapy will be discontinued from the study medication for any one of the following reasons:

- Observation of rapid enlargement of the lesion despite drug treatment, and not attributed to a paradoxical response.
- Presence of SAE or adverse drug reaction of NIH/NCI Common Toxicity Criteria [33] grade 3 or 4 that prevent adherence to treatment.
- Need to use any drug with anti-mycobacterial activity or likely to interfere with interpretation of the results during the treatment period.
- Allergic reaction to a study drug.
- Observation of any new condition or situation (including pregnancy, major trauma, or severe immune compromise) developing during the trial, which in the judgement of the investigation team, may interfere with continued participation in the study.
- At the request of the subject / withdrawal of consent or assent.
- Non-compliance with scheduled follow-up visits.
- Non-compliance with medication.
- If the investigator considers that a patient’s health will be compromised due to AEs, SAEs, or a concomitant illness that develops after entering the study.

They will however be followed up for safety and included in the analysis.

#### Procedures for withdrawal from the study

Subjects withdrawn from the study will not be replaced (5% drop out has been assumed for power calculations) but will be included in the analysis. Subjects who withdraw will be invited to continue attending study clinics for routine care and measurements and will be offered the standard of care according to the PNLLUB guidelines. Reasons for withdrawal will be recorded in the CRF and in the subjects’ medical notes and will be taken into account to prevent any potential loss of patients.

### Pharmacovigilance

Safety of the two treatment regimens will be assessed through routine monitoring of AEs. At each of the scheduled follow-up visits, patients will be enquired about current AEs or any events observed during the period prior to the visit. In addition, evaluation of haematology and blood chemistry parameters, performing ECG, regular measurement of vital signs, and physical examinations will be made at scheduled follow-up according to the visit schedule. AEs could also be detected when reported by the patient during or between visits. At randomization, patients will be given two telephone numbers, which they can call or send a text message/WhatsApp in the event of experiencing an AE. All AEs causing discontinuation, interruption, or adjustment of the trial therapy must be recorded in the AE section of the CRF, regardless of the assumption of a causal relationship. Documentation of AEs includes date of onset and offset, pattern, duration, intensity, impact, actions taken, severity, and outcome.

The Clinical PI should also evaluate the probability of a causal relationship of the AE to the trial medication. Any patient who develops an AE will be followed up and treated appropriately until he/she returns to normal condition. Any treatment, if necessary, will be recorded in the CRF.

All AEs (including laboratory abnormalities) will be recorded in the CRFs. After the trial has been completed or terminated, all recorded AEs will be listed, evaluated, and discussed in the final report.

Furthermore, in order to ensure the safety of the patient, from the moment that informed consent has been obtained from the patient and until 30 days after the patient has discontinued from the study drugs, any AE must be reported immediately to the site study coordinator and the site PI and to the Data Safety and Monitoring Board (DSMB) within 24 h of patient’s notification or identification of the occurrence by the clinical staff, regardless of suspected causality.

Any SAE must be reported to the DSMB, the Benin Ethics Committee (EC), and the Sponsor within 24 h of patient’s notification or identification of the occurrence by the clinical staff, regardless of suspected causality.

### Endpoints of the study

#### Definitions of the populations for analysis

- Intention-to-Treat Exposed (ITT-E): this population will consist of all randomized patients who receive at least one dose of randomized study medication. Patients will be assessed according to their randomized treatment, regardless of the treatment they receive.
- Per Protocol (PP): this population will consist of subjects in the ITT-E population who complete the study and are not major protocol violators.
- Per Protocol (PP) PCR-positive population: The PP PCR-positive population includes those randomized patients with a clinical diagnosis of very likely BU or likely BU, PCR-positive, and with no major violations of the protocol. Unless stated otherwise, the PP PCR-positive population will be used for efficacy analyses.
- Intention-to-Treat Exposed (ITT-E) PCR-positive population: The ITT-E PCR-positive population includes those randomized patients with a clinical diagnosis of very likely BU or likely BU, PCR-positive that have, at least, taken one dose of the study drugs. This population might include major violators of the protocol. The ITT-E PCR-positive population will be also used for secondary analysis of efficacy.
- Per Protocol (PP) Clinical Diagnosed (CD) population: The PP CD population includes those randomized patients with a clinical diagnosis of very likely BU or likely BU and with no major violations of the protocol. This population includes both PCR-positive and PCR-negative. The PP CD population will be also used for secondary analysis of efficacy.
- Intention-to-Treat Exposed (ITT-E) Clinical Diagnose (CD) population: The ITT-E CD population includes those randomized patients with a clinical diagnosis of very likely BU or likely BU that have, at least, taken one dose of the study drugs. This population might include both PCR-positive and PCR-negative and major violators of the protocol. The ITT-E CD population will be also used for secondary analysis of efficacy.
- Safety: The Safety Population (SP) is defined as all subjects who receive at least one dose of randomized study medication. Subjects will be analysed according to the actual treatments received. Unless otherwise stated, the SP will be used for safety analyses.
- Pharmacokinetic (PK): the PK population is defined as the participants in the SP who receive at least one dose of randomized study medication and have at least one evaluable PK sample. Participants will be analysed according to the treatment actually received.

#### Primary endpoint

The primary clinical efficacy endpoint is cure rate, i.e. proportion of patients with complete lesion healing without recurrence and without excision surgery 12 months after treatment initiation, in the PP PCR-positive population.

#### Secondary endpoints

Secondary endpoints include:

1. Derive and compare main PK parameters for RIF, CLA, and AMX for the two groups (RC8 and RCA4) at a steady state. These include area under the curve (AUC), trough concentration (*C*_*τ*_), maximum observed drug concentration (*C*_max_), time to maximum observed drug concentration (*t*_max_), and elimination half-life (*t*_1/2_).
2. Characterize the POPPK in BU patients randomized on RCA4 and derive population PK parameters of interest, such as apparent Clearance (CL/F), apparent volume of distribution (V/F), and absorption rate (Ka), together with potential covariates of interest. This will involve investigating inter- and intra-subject variability for RIF and AMX.
3. Rate of complete lesion healing without recurrence and without excision surgery, 12 months after start of treatment in the ITT-E PCR-positive, PP CD, and ITT-E CD populations.
4. Rate of complete lesion healing without recurrence and without excision surgery 12 months after start of treatment by category (I, II, and III) lesions analysis in all ITT-E and PP populations.
5. Recurrence rate within 12 months of treatment initiation in all ITT-E and PP populations.
6. Treatment discontinuation rate in all ITT-E and PP populations.
7. Treatment compliance rate in all ITT-E and PP populations.
8. Rate of paradoxical response within 12 months of treatment initiation in all ITT-E and PP populations.
9. Median time to healing after treatment initiation in all ITT-E and PP populations.
10. Proportion of patients with reduction in lesion surface area within 12 months of treatment initiation in all ITT-E and PP populations.
11. Interval between healing and recurrence within 12 months of treatment initiation in all ITT-E and PP populations.
12. Incidence of all AEs, SAEs, and serious unexpected suspected adverse drug reactions (SUSAR) within 12 months of treatment initiation among treatment arms in all SP, ITT-E, and PP populations.
13. Rate of median bacterial clearance among treatment arms in the bacterial clearance sub-study population.
14. Rate of patients with BUFLS improvement within 12 months of treatment initiation among treatment arms in all ITT-E and PP populations.

### Data management

#### Data collection and management

Source documents include subject hospital/clinic records, physician’s and nurse’s notes, appointment book, original laboratory reports, digital pictures of wound lesions, and special assessment reports, signed informed consent forms, and subject screening and enrolment logs. In order to ensure data quality, a uniform hard copy CRF (in French) will be designed for use at all the study sites. A CRF for each randomized patient will be completed at each study visit.

Study site staff and the PI in each site will be responsible for evaluating the CRFs for completeness before entering into an electronic clinical database. Once entered, data will be collected and sent on an ongoing basis to the DMMC for cleaning. The central electronic database will be based on the Open Data Kit platform, with data encrypted in transit and at rest in ODK Cloud, where data are backed up continuously [https://getodk.org/]. The data manager will review the data for discrepancies and missing data. The site will then be informed to make any required corrections and/or additions. Moreover, CRFs and source documents will be monitored by the Monitoring Team (MT) (ISCIII). Discrepancies noted either by the monitor or data centre will be queried to the site PI. The trial data will be stored in a computer database maintaining confidentiality in accordance with national data legislation and Good Clinical Practice (GCP). The data management plan will outline the procedures and guidelines to ensure data quality.

Patient information will be stored in a high-security computer system and kept strictly confidential. Confidentiality will be ensured utilizing a patient identification code number corresponding to treatment data on the computerized files.

#### Access to data, monitoring, and audits

Monitoring visits to the trial site will be made periodically by the sponsor representatives and/or designated monitors to ensure that GCPs and all aspects of the study protocol are being followed. The monitoring visits will provide the opportunity to evaluate the progress of the study, verify the accuracy and completeness of CRFs/SAE forms, resolve any inconsistencies in the study records, and ensure that all protocol requirements, applicable regulations, and investigator’s obligations are being fulfilled. At least four types of visits are planned: pre-study, study start, during the study, and study end. During the actual recruitment period (years 2 and 3 of the study), visits will be performed at least twice a year.

Source documents and SAE forms will be reviewed for verification of consistency with data on CRFs. Site investigators will facilitate direct access to all CRFs/SAE forms, medical records, laboratory work sheets and to assess the status of drug storage, dispensing, and retrieval at any time during the study. The corresponding source documents for each subject will be made available provided that subject confidentiality is maintained in accord with local regulations.

It will be the monitors’ responsibility to inspect the CRFs/SAE forms at regular intervals throughout the study, to verify the adherence to the protocol and the completeness, consistency, and accuracy of the data being entered on them.

### Statistical analysis

#### General considerations

The rationale for selecting the non-inferiority study design is that, considering the efficacy of the standard treatment (RC8) of as high as 95.9% (first quartile–third quartile 91.3–98.5%) [34] (*ClinicalTrials.gov Identifier: NCT01659437*) or 96% in a 14-week delayed-decision to surgery group [32] (*ClinicalTrials.gov identifier: NCT01432925*), whether the experimental treatment (RCA4) could be equal or better is not the relevant question. Rather, the main consideration is to what extent shortening the treatment to 4 weeks with RCA4 could be inferior to the current standard of care.

#### Sample size

The sample size calculation is based on clinically meaningful non-inferiority between the standard treatment group (RC8) and the investigational treatment group (RCA4), as suggested by key informants, experts in BU case management [34] (*ClinicalTrials.gov Identifier: NCT01659437*); [32] (*ClinicalTrials.gov identifier: NCT01432925*). Assumptions are:

- Equal cure rate of 95.9% for PCR-confirmed patients the two arms (RC8 & RCA4).
 − 10% non-inferiority margin.
- 2.5% one-sided alpha level.

Under these assumptions, the study would require 62 PCR-confirmed patients per treatment arm to reach 80% power (total sample size = 124). It is estimated that 5% of PCR-confirmed patients will be lost-to-follow-up, so 66 PCR-confirmed patients are needed in each arm (or 132 in total) to achieve the required 62 evaluable patients. Pobè treatment centre reports a PCR confirmation rate of approximately 95% in patients clinically diagnosed as very likely or likely to have BU following the WHO scoring criteria. Therefore, approximately 70 clinically diagnosed BU patients will need to be recruited in each arm (or approximately 140 in total) to reach the 66 PCR-confirmed. Recruitment will continue until at least 66 PCR-confirmed patients have been randomized to each arm.

Recruitment rates will be monitored closely and, if it is feasible within the timeframe available, the study will be extended to achieve 90% power. For 90% power, based on the assumptions above, the study would require data from 83 evaluable PCR-confirmed patients per treatment arm (or 166 in total). Allowing for 5% lost-to-follow-up means 88 PCR-confirmed patients are needed in each arm (or 176 in total). To achieve this, approximately 93 clinically diagnosed BU patients will need to be recruited in each arm (or approximately 186 in total). In this scenario, recruitment will continue until at least 88 PCR-confirmed patients have been randomized to each arm.

#### Efficacy analysis

Primary efficacy analysis will be determined for the PP PCR-positive population. The adjusted estimate of the difference (expressed as an absolute change and not a relative change) in the cure rate between the two arms will be presented along with 95% two-sided CI based on a stratified analysis using Cochran-Mantel–Haenszel (CMH) weights. For the statistical analysis, BU categories I-III will be the three strata. The difference in cure rate between the two treatment groups (RC8 *vs.* RCA4) and the corresponding 95% CI will be calculated. Treatment with RCA4 will be declared non-inferior to treatment with RC8 if the lower bound of a two-sided 95% confidence interval (CI) for the difference in cure rates (expressed as an absolute change and not a relative change) between the two treatment arms is greater than − 10%. A superiority hypothesis will be tested once non-inferiority is proven. Superiority can be concluded if the lower bound of a two-sided 95% confidence interval for the difference in cure rates (expressed as an absolute change and not a relative change) between the two treatment arms is greater than 0%. Details for analysis of secondary efficacy outcomes will be included in the Statistical Analysis Plan (SAP).

#### Analysis of missing outcome data

The investigators will describe the reason why patients were lost to follow-up and, hence, why outcomes were missing. To identify the potential for bias due to missing data, participants’ characteristics with and without missing values will be compared. A “baseline table” of prognostic characteristics will be presented, to assess whether these characteristics are indeed balanced between the treatment groups immediately after randomization. A “second baseline table” with a comparison of prognostic baseline characteristics between the participants in the study groups who are actually included in the analysis (i.e. those with observed outcomes) will be also prepared, showing the distribution of baseline characteristics among the treatment groups for patients for whom outcomes were observed and who were included in the analysis. Finally, imputation of missing data will be carried out subsequently for sensitivity analyses using the multiple imputation technique.

#### Safety analysis

All safety endpoints will be summarized based on the SP. Exposure to randomized study medication (measured by the number of days on study drug) will be summarized by treatment group. The proportion of subjects reporting AEs and SAEs will be tabulated for each treatment group.

#### Interim analysis

For many clinical trials, interim analyses are undertaken periodically to determine whether to stop the trial early because of a substantial treatment difference or futility. However, in the field of BU, the lack of accurate and reliable early biomarkers of BU cure requires a 12-month follow-up of patients to assess cure; thus, considering the small sample size and short timelines of the project, an interim analysis will not be feasible. As such, no interim analysis will be performed.

### Pharmacokinetic study

The pharmacokinetic interactions between RIF and CLA in BU patients were investigated by Alffeenaar et al. [35]. A descriptive PK study will be conducted within this clinical trial in order to explore potential pharmacokinetic interactions between AMX/CLV and RIF/CLA.

### Effect of AMX/CLV on RIF/CLA

The potential effects of AMX/CLV on RIF/CLA’s will be investigated at steady-state through the comparison of the derived PK parameters in the two groups in a subset of 16 BU patients (8 randomized in the RC8 group and 8 in the investigational treatment group RCA4) will be included in the PK study. Due to safety reasons, only inpatients aged ≥ 15 years old and weight > 40 kg, who are part of the main clinical trial in the Pobè treatment centre, will be included in chronological order. The only specific exclusion criteria will be a creatinine level higher than the normal one in Benin (7–14 mg/L).

Blood samples of approximately 3–4 mL will be collected once at time 0 (within 10 min pre-dose) and at times 0.5, 1, 1.5, 2, 2.5, 3, 5, 7.5, and 10 h post-dose, prior to the second daily dose of CLA and AMX/CLV, during the window between days 7 (week 1) and 14 (week 2) after starting the treatment. Samples will be stored at − 20 or − 80 °C during a particular time interval according to the Contract Research Organization´s (CRO) specifications. Then, they will be sent to the CRO for bioanalysis by liquid chromatography-tandem mass spectrometry (LC/MS/MS).

Plasma concentrations of RIF, CLA, and AMX will be analysed by non-compartmental analysis with Phoenix WinNonlin 8.2 or higher. Several PK parameters, including area under the plasma concentration–time curve [AUC0 − *τ*, AUC0 − 10] at steady-state, *C*_*τ*_, *C*_max_, *t*_max_, and *t*_1/2_, will be determined for RIF and CLA in the RC8 group and for RIF, CLA, and AMX in the RCA4 group as data permit. Furthermore, an exploratory statistical analysis will investigate any potential impact on the PK of RIF/CLA in the presence of AMX/CLV.

### POPPK analysis

A POPPK analysis will be conducted to derive population PK parameters of interest (i.e. CL/F and V/F, and Ka) for RIF and AMX (RCA4 group) and to investigate potential covariates of interest. Covariate effects (mainly patients’ demographics and key lab measurements) will be considered primarily for PK parameters (CL/F and V/F) and may be explored for a limited number of other parameters (Ka) as appropriate and depending upon the final form of the structural model and data availability. Both inter- and intra-subject variability will be considered in this analysis.

### Bacterial clearance study

This study will be performed in order to test the hypothesis that a faster bacterial clearance rate of RCA4 correlates to a shorter median time to healing. Three FNA or swabs will be taken from unhealed lesions at the time of initiation of treatment (baseline, week 0) and at weeks 1, 2, 4, 8, and 14 after treatment initiation. DNA/RNA will be extracted from samples and a combined 16S rRNA reverse transcriptase/ IS2404 qPCR assay will be performed to detect viable bacteria [36, 37]. Healing and bacterial clearance may be different according to the lesion category. Therefore, sample size will be *n* = 54, which means 27 patients per treatment arm stratified by category I, II, and III (9 per category). Only patients who are part of the main clinical trial in the Pobè treatment centre will be included in chronological order according to arm and lesion category.

### Committees for the study

The Steering Committee (SC) consists of a representative from each partner and will be responsible for the overall execution of the action, mainly ensuring alignment and coordination of the activities. The Trial Management Committee (TMC), which consists of the clinical PI, the leading physicians of the three clinical sites, the local manager, and the Project Coordinating Office manager in Benin, will be responsible of the trial field logistics and will address the operational matters. The MT will ensure that GCPs and all aspects of the protocol are followed. Partner ISCIII will be responsible for the monitoring activities and representatives from the other partners will be part of this committee. At least one monitoring visit will be performed every year.

In addition, three international independent boards have been created:

- Data Safety and Monitoring Board (DSMB). This group of experts will advise the BLMs4BU Consortium for safety and efficacy. The DSMB will monitor the study in order to ensure that harm is minimized and benefits maximized for the study subject.
- Independent Advisory Board (IAB). IAB will advise the project as a whole on scientific and technical development.
- Technical Expert Panel (TEP). During the clinical follow-up, pictures of the lesions are taken systematically at every scheduled visit to document clinical evolution. On a regular basis (every 4 months) and when each patient reaches the 14 weeks visit, these pictures will be sent to the TEP, who are a clinical expert panel of BU physicians. They will be blinded to treatment allocation and visit at which pictures have been taken. This panel of experts will be completely different from those treating patients in each study site and will give an independent opinion on whether or not to use surgery as in previous clinical trials and to assess healing and cure.

## Discussion

The new WHO Road Map for NTDs (2021–2030) calls for innovative approaches to address a new set of targets. For BU, one of the needed actions is to evaluate new medicines or regimens (ideally by oral administration) to reduce treatment duration [38]. Building on these needs, the BLMs4BU study is a randomized and open clinical trial that aims to assess the possibility to shorten BU treatment. In many BU endemic countries, antibiotic treatment is free of charge. However, other expenditures and efforts prevent patients from seeking care at the formal health system. The economic burden arising from transport, accommodation and food for patients and caregivers, lost earnings, and work force is too high for the affected families, in particular if long hospital stays are required. These expenses affect the health system and the patient/family/community. A shortened, highly effective, all-oral regimen would potentially improve care of BU patients by reducing duration of treatment and time to healing for all type of lesions. Furthermore, this would have an impact on hospitalization-related expenses (fewer hospital days), indirect costs (loss of income and family costs to support the patient), and barriers to access therapy (easier and shorter therapy).

Although our study is limited to Benin, this study will create a new paradigm for better BU treatment, i.e. intensified therapy for a shorter period. Moreover, it will provide evidence to expand it to other endemic countries, eventually facilitating broad implementation of the new regimen and informing WHO policy decision making in the field of BU therapy.

This intervention will also have a transversal approach that involves community engagement, awareness campaigns, and capacity building activities. Skin NTDs are often co-endemic in many countries, districts, and communities. Thus, our study presents an opportunity to build on integration, which both will increase cost-effectiveness and expand coverage to fight other skin NTDs.

This trial might also provide insightful information on treatment shortening strategies for other mycobacterial infections having RIF as the cornerstone, including TB or leprosy, and other non-tuberculous mycobacteria infections. In fact, since TB clinical trials addressing treatment shortening might need up to 3 years of patient follow-up to assess relapse of the disease, the much faster design of BU clinical trials could offer quick proof-of-concept data to be translated to TB therapy.

### Trial status

The study was registered only once in ClinicalTrials.gov (Ref. NCT05169554) on 27 December 2021. The recruitment started in December 2021 and is meant to last until November 2023, with duration of recruitment of 2 years.

## Supplementary Information

**Additional file 1:** SPIRIT checklist for the BLMs4BU study.

## Data Availability

Individual participant data after de-identification and trial tools including study protocol, statistical analysis plan, and analytic code will be made available 1 month after publication of the article reporting the main findings of this trial, with no end date, to researchers who provide a methodologically sound proposal, upon signature of a data access agreement. Proposals should be directed to the corresponding author.

## Abbreviations

AE: Adverse event
AFB: Acid-fast bacilli
AMX/CLV: Amoxicillin / clavulanate
ARAID: Research and Development Agency of Aragon Foundation
AUC: Area under the curve
BLMs4BU: Beta-lactams for Buruli ulcer
BU: Buruli ulcer
BUFLS: Buruli ulcer functional limitation score
CDTUB: Centers for Detection and Treatment of Buruli ulcer
*C*_*τ*_: Through concentration
CLA: Clarithromycin
CL/F: Apparent clearance
*C*_max_: Maximum observed drug concentration
CMH: Cochran-Mantel–Haenszel
CRF: Case report form
CI: Confidence interval
CRO: Contract research organization
DMMC: Data management and monitoring centre
DSMB: Data safety and monitoring board
EC: Ethics committee
ECG: Electrocardiogram
FNA: Fine-needle aspiration
GCP: Good clinical practice
hCG: Human chorionic gonadotropin
HIV: Human immunodeficiency virus
IAB: Independent advisory board
ICMJE: International Committee of Medical Journal Editors
IRB: Institutional Review Board
ISCIII: Instituto de Salud Carlos III
ITT-E: Intention-to-Treat Exposed population
ITT-E CD: Intention-to-Treat Exposed Clinical Diagnose population
ITT-E PCR-positive: Intention-to-Treat Exposed PCR-positive population
Ka: Absorption rate
LPLV: Last patient, last visit
LC/MS/MS: Liquid chromatography-tandem mass spectrometry
MT: Monitoring team
NTD: Neglected tropical disease
pC: Expected proportion of the outcome of interest in the control group
PI: Principal investigator
PK: Pharmacokinetic
POPPK: Population pharmacokinetic
PNLLUB: BU National Program
PP: Per Protocol population
PP CD: Per Protocol Clinical Diagnose population
PP PCR-positive: Per Protocol PCR-positive population
pT: Expected proportion of the outcome of interest in the treatment group
qPCR: Quantitative polymerase chain reaction
QTc: Corrected QT
RC8: 8 Weeks rifampicin plus clarithromycin
RCA4: 4 Weeks rifampicin plus clarithromycin plus amoxicillin/clavulanate
RIF: Rifampicin
SAE: Serious adverse events
SAP: Statistical Analysis Plan
SC: Steering Committee
SP: Safety Population
SUSAR: Serious unexpected suspected adverse drug reactions
TB: Tuberculosis
TCOLF: Tres Cantos Open Lab Foundation
TEP: Technical expert panel
*t*_max_: Time to maximum observed drug concentration
TMC: Trial Management Committee
V/F: Apparent volume of distribution
WHO: World Health Organization
